# HDR prostate brachytherapy plan robustness and its effect on in‐vivo source tracking error thresholds: A multi‐institutional study

**DOI:** 10.1002/mp.15658

**Published:** 2022-04-19

**Authors:** Joel Poder, Dylan Koprivec, Yashiv Dookie, Andrew Howie, Dean Cutajar, Antonio L. Damato, Nicolas Côté, Marco Petasecca, Joseph Bucci, Anatoly Rosenfeld

**Affiliations:** ^1^ Department of Radiation Oncology St George Cancer Care Centre Kogarah New South Wales Australia; ^2^ Centre for Medical Radiation Physics, University of Wollongong Wollongong New South Wales Australia; ^3^ Department of Medical Physics, Memorial Sloan Kettering Cancer Centre New York New York USA

**Keywords:** brachytherapy, HDR, in‐vivo, prostate, robustness, source tracking

## Abstract

**Purpose:**

The purpose of this study was to examine the effect of departmental planning techniques on appropriate in‐vivo source tracking error thresholds for high dose rate (HDR) prostate brachytherapy (BT) treatments, and to determine if a single in‐vivo source tracking error threshold would be appropriate for the same patient anatomy.

**Methods:**

The prostate, rectum, and urethra were contoured on a single patient transrectal ultrasound (TRUS) dataset. Anonymized DICOM files were disseminated to 16 departments who created an HDR prostate BT treatment plan on the dataset with a prescription dose of 15 Gy in a single fraction. Departments were asked to follow their own local treatment planning guidelines. Source positioning errors were then simulated in the 16 treatment plans and the effect on dose–volume histogram (DVH) indices calculated. Change in DVH indices were used to determine appropriate in‐vivo source tracking error thresholds. Plans were considered to require intervention if the following DVH conditions occurred: prostate V100% < 90%, urethra D0.1cc > 118%, and rectumtt *D*max > 80%.

**Results:**

There was wide variation in appropriate in‐vivo source tracking error thresholds among the 16 participating departments, ranging from 1 to 6 mm. Appropriate in‐vivo source tracking error thresholds were also found to depend on the direction of the source positioning error and the endpoint. A robustness parameter was derived, and found to correlate with the sensitivity of plans to source positioning errors.

**Conclusions:**

A single HDR prostate BT in‐vivo source tracking error threshold cannot be applied across multiple departments, even for the same patient anatomy. The burden on in‐vivo source tracking devices may be eased through improving HDR prostate BT plan robustness during the plan optimisation phase.

## 1 INTRODUCTION

High dose rate brachytherapy (HDR BT) has been proven to be an effective modality for the treatment of prostate cancer, whether used as a monotherapy or in combination of external beam radiation therapy (EBRT).^1^, ^2^, ^3^ The effectiveness of the treatment however, may depend strongly on the ability to precisely position the HDR BT source (typically Ir‐192), relative to the anatomy of the patient, in the same arrangement from the BT treatment planning systems (BTPSs). Deviations in planned vs. delivered dwell positions of only a few millimeters may result in a decrease in tumor control probability and/or an increase in normal tissue complication probability.^4^, ^5^

There has been a substantial increase in the effort toward more technologically advanced in‐vivo treatment verification in recent years, particularly for application in HDR prostate BT.^6^, ^7^ The predominant aim of these studies has been to detect and minimize the occurrence of discrepancies between planned and measured HDR prostate BT dwell positions. Much of this technological evolution has been focused on the development of novel radiation detectors and detector arrays for the purposes of in‐vivo source tracking.^8^, ^9^, ^10^, ^11^, ^12^ The advantage of in‐vivo source tracking over traditional point‐based in‐vivo dosimetry is the ability to determine actually delivered (within measurement uncertainty) dwell positions in three‐dimensional (3D) coordinates, and also measure dwell times.^8^, ^10^, ^12^ When combined with real‐time imaging, in‐vivo source tracking may allow for a “delivered” 3D dose distribution to be reconstructed, giving more clinically meaningful results.^6^

While there has been significant development in detector systems to perform in‐vivo source tracking in HDR prostate BT, there have been minimal published studies focusing on the minimal discrepancy between planned and delivered dwell positions that must be resolved by in‐vivo source tracking devices to detect clinically significant changes in dose distributions. One previous study examined the influence of patient anatomical differences on appropriate in‐vivo source tracking error thresholds by simulating source positioning errors in 20 retrospective HDR prostate BT treatment plans.^13^ The study found that appropriate source positioning error thresholds varied between 2–5 mm and were dependent on the direction of the source positioning error, the position relative to the anatomy, and the weight of the dwell positions that were simulated with the source positioning error.

The purpose of this study was to examine the effect of departmental planning techniques on appropriate in‐vivo source tracking error thresholds and determine if a single in‐vivo source tracking error threshold would be appropriate for the same patient anatomy.

## 2 MATERIALS AND METHODS

### 2.1 Patient anatomical data and multi‐institutional plans

A set of trans‐rectal ultrasound (TRUS) images and corresponding structure set from a patient who had previously been treated with a TRUS‐based HDR prostate BT boost at St. George Cancer Care Centre (STGCCC), Sydney, Australia, were randomly selected for use in this study. At STGCCC, TRUS‐based HDR prostate BT is planned using the Oncentra Prostate BTPS (v4.6, Elekta BT, Veenendaal, the Netherlands) and prescribed a boost dose of 15 Gy in a single fraction. Details of the TRUS‐based HDR prostate BT boost technique at STGCCC can be found in a previous publication.^13^

At STGCCC, TRUS images obtained for the purposes of HDR prostate BT treatment planning are acquired by first covering the TRUS probe (Endocavity Biplane E14CL4b, BK Medical, Peabody, MA, USA) with an endorectal balloon, and then inserting the TRUS probe within the patient placed in the dorsal lithotomy position. A 6‐mm Foley catheter is inserted prior to TRUS image acquisition to aid in visualization of the urethra on the TRUS images. After positioning of the probe to obtain a clear image of the prostate and surrounding anatomy and prior to inserting any BT needles, a 3D ultrasound image is acquired via sagittal rotation of the TRUS probe, resulting in an image resolution of 0.5 × 0.5 × 0.5 mm^3^. A radiation oncologist then contours the prostate, urethra, and rectum on the 3D ultrasound image set. Contours of the rectum and urethra are extended 10 mm caudally from the apex and 10 mm cranially from the base of the prostate. The urethra contour contains the bladder neck, based on the ultrasound visible filled part of the bladder.

The patient dataset used in this study contained a prostate contour of 35 cubic centimeters (cc). The DICOM ultrasound images and structure set were anonymized and exported from oncentra prostate. The anonymized files were then shared with 15 additional departments located in Australia, Europe, the United Kingdom, and North America who agreed to participate in this study. Participating departments were asked to import the TRUS images and structure set files into their local TRUS‐based HDR prostate BT treatment planning system (BTPS) and create a treatment plan with a 15 Gy prescription according to their own local treatment planning guidelines. Catheter locations dwell position/dwell weight optimization, and prioritization of target coverage versus OAR dose limits were all determined by each department individually, according to local protocols. The DICOM file containing the BT treatment plan was then exported from the department's BTPS and returned to STGCCC for analysis.

### 2.2 Simulated treatment planning source positioning errors

To manually simulate source positioning errors in the 16 HDR prostate BT treatment plans used in this study, a script was developed in Python to edit the 3D coordinates of the dwell positions in the treatment plans. As identified in a previous study, the two distinct types of treatment source positioning errors were considered in this study.^14^

The first type of source positioning error results in delivered dwell positions of *all* catheters in the plan being shifted in the cranial/caudal direction relative to their planned positions. Examples of these types of errors include incorrect catheter index length used in BTPS, BTPS coordinate system origin not set correctly, and incorrect catheter free length used in BTPS.^14^ In this study, these source positioning errors were simulated by shifting dwell positions in all catheters from ‐6 mm (caudal) to +6 mm (cranial) in 1 mm increments.

The second type of source positioning error that can be considered results in dwell positions of *some* catheters being shifted in the anterior/posterior, or medial/lateral directions relative to their planned positions. Examples of these types of errors include catheter reconstruction errors and incorrect grid position being selected for catheter reconstruction.^14^ Source positioning errors of this type were again simulated from ‐6 mm (posterior/medial) to +6 mm (anterior/lateral) in 1 mm increments. As a previous study by Rylander et al. identified a median of three catheters per plan requiring correction in HDR prostate BT,^15^ three catheters per plan were considered for error simulation of this type in this current study. Therefore, a somewhat worst‐case scenario was considered, with source positioning errors from the three most heavily weighted catheters (the three catheters with the largest total dwell time) in the plan occurring in the same direction, that is, all dwell positions in the three most heavily weighted catheters moved medially, laterally, anteriorly, or posteriorly.

### 2.3 Dose volume histogram evaluation

Plans from all 16 departments were assessed by evaluating the dose volume histogram (DVH) indices shown in Table 1. These DVH indices have been shown to produce HDR prostate BT boost plans for 15 Gy prescriptions that result in acceptable short‐ and medium‐term toxicities and quality of life.^1^ For each department's plans, the change in DVH index was calculated as a function of the magnitude of the source positioning error.

**TABLE 1:** Dose volume histogram criteria used to assess plan acceptability

Structure	DVH Parameter	Acceptable (%)
Prostate	V_100_%	>95
Urethra	D_0.1cc_	≤118
Rectum	D_max_	≤80

Prostate V_100_(%) is the volume of prostate (in percent) being irradiated by the 100% isodose line. Urethra D_0.1cc_ is the dose to the most highly irradiated 0.1cc of urethral tissue. Rectum *D*max is the maximum point dose to the rectum reported by the brachytherapy treatment planning system.

A study by Hoskin et al., found that for the prostate, both the D90% (the dose received by 90% of the prostate volume) and the V100% (volume of prostate being irradiated by the 100% isodose) were significant predictors for biochemical relapse in intermediate‐ and high‐risk patients treated with a HDR prostate BT boost. The study showed that a 5% decrease in either D90% or V100% corresponded to a 10% decrease in biochemical control.^16^ As a V100% > 95% was considered acceptable in this study, a V100% < 90% was used as an action threshold. For the OAR constraints in Table 1, an action threshold was set for any time, a source positioning error resulted in a violation of these constraints.

### 2.4 Plan robustness parameter

In an attempt to quantitatively describe the robustness of each plan to source positioning errors, a *plan robustness parameter* was derived. The equation derived to calculate the plan robustness parameter is shown below (Equation 1), where *w* is the relative weight of the dwell position in the total plan, *d* is the 3D vector length from the center of the dwell position to the nearest point on the surface of the contour of interest (prostate, rectum, or urethra) in millimeter, and *n* is the number of dwell positions in the plan. The plan robustness parameter is considered separately for each contour in the plan. A smaller robustness parameter corresponds to more lightly weighted dwell positions at larger distances from the contour surface, and therefore, should be more robust to source positioning errors. Conversely, a larger robustness parameter corresponds to more heavily weighted dwell positions at smaller distances from the contour surface, and should be less robust to source positioning errors. The BT plan robustness parameter was calculated for each of the contours separately, for each of the 16 treatment plans used in this study.

(1) $$\text{Robustness}\;\text{Parameter}\;=\sum {}_{i=1}^{n}\frac{w_{i}}{d_{i}^{2}}.$$

## 3 RESULTS

### 3.1 Initial treatment plan characteristics

Characteristics of the 16 initial treatment plans generated as part of this study are shown in Table 2. Four of the 16 participating departments used the Varian Vitesse real‐time HDR prostate BTPS and 12 of the 16 departments used the Elekta Oncentra BT BTPS. All departments using the Varian Vitesse system used a source step size of 5 mm, and all departments using the Elekta Oncentra BT system used a source step size of 1 mm. Participating departments used a combination of inverse, graphical, and manual optimization as part of their treatment planning process.

**TABLE 2:** Characteristics of the 16 initial departmental treatment plans generated as part of this study

Characteristics	Average	Standard Deviation
Prostate V_100_%	97.2%	2.2%
Urethra D_0.1cc_	112.1%	1.6%
Rectum *D*_max_	77.2%	1.7%
Number of catheters	16	2
Number of dwell positions	186	71
Total reference air kerma (cGycm^2^)	4063	406

The variation in step size used across the two BTPSs explains the wide variation in the number of dwell positions used across the 16 plans. All but one department met the prostate V100% criteria outlined in Table 1, and all departments are able to successfully meet the urethra and rectum constraints in their initial plans.

**FIGURE 1:**
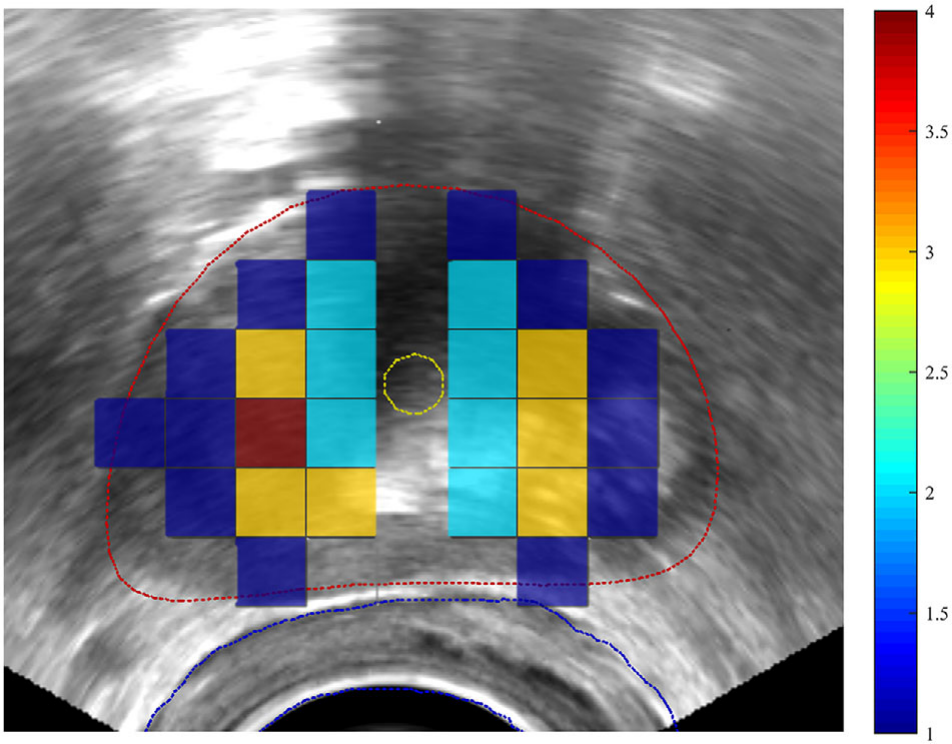
Axial TRUS slice of the patient anatomy, the prostate, urethra, and rectum contours are shown in red, yellow, and blue, respectively. The frequency of location of the three most heavily weighted catheters across all the plans is shown using the colour scale

The frequency of location of the three most heavily weighted catheters across all plans on a representative axial slice of the patient anatomy is shown in Figure 1. As can be seen from the figure, the three most heavily weighted catheters are most frequently positioned within the peripheral zone of the prostate.

### 3.2 Effect of source positioning errors on dose–volume histogram indices

**FIGURE 2:**
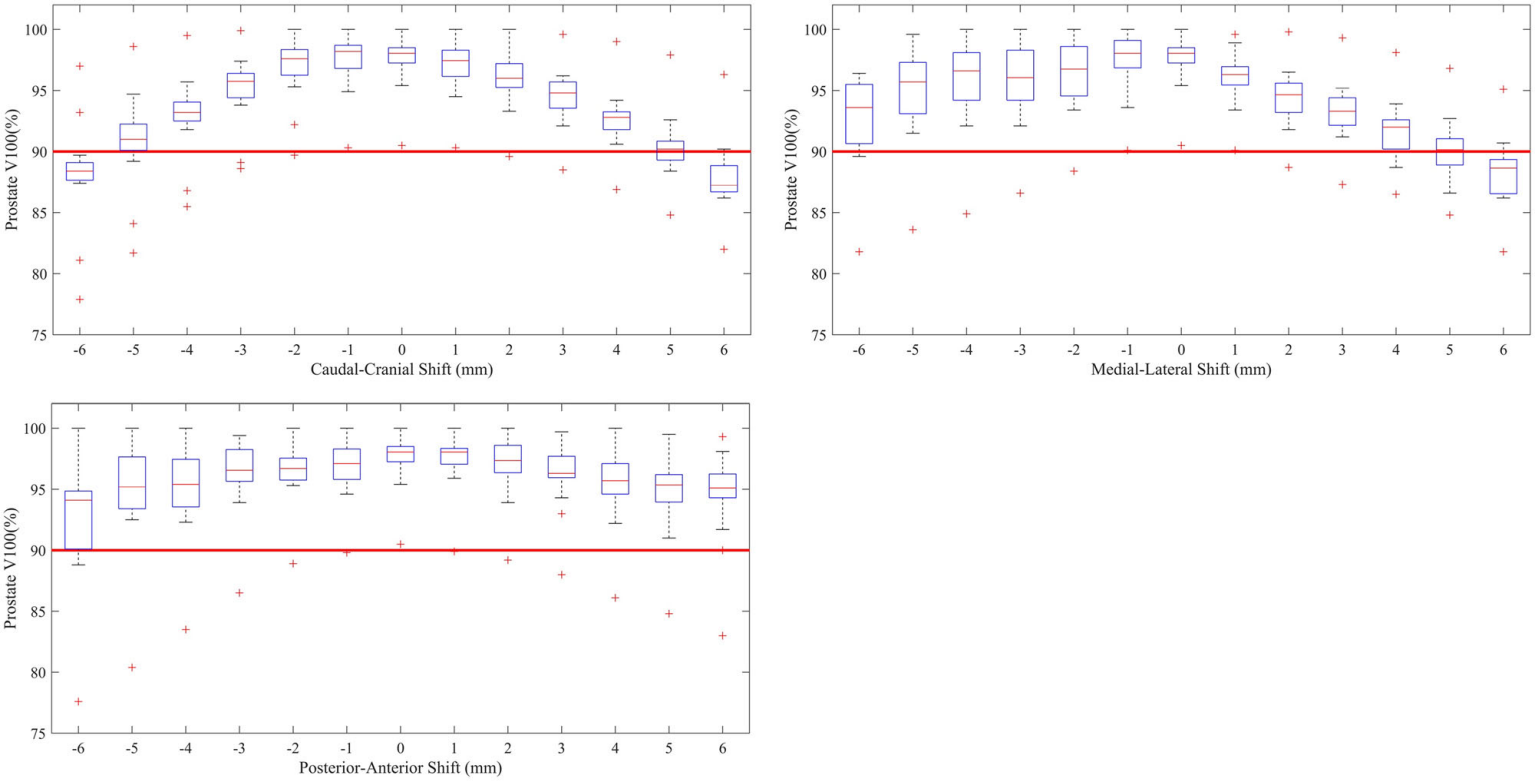
(A) Change in prostate V100% DVH metric as a function of source positioning error in the caudal (negative) and cranial (positive) direction, (B) the medial (negative) and lateral (positive) direction, and, (C) the posterior (negative) and anterior (positive) direction. Red lines within the boxes represent the median across all 16 departments, the boxes represent the interquartile range, whiskers represent the minimum and maximum, red crosses represent the outliers. The thick solid red line represents the tolerance of 90% coverage for the prostate V100% used to determine the source tracking error threshold

The effect of source positioning errors on the prostate V100% is shown in Figure 2. As can be seen from the differences in Figure 2A‐C, the effect of source positioning errors in the prostate V100% is directionally dependent, with shifts in the caudal‐cranial direction being more sensitive than those in the posterior–anterior and medial–lateral directions. Even more pertinent is the significant spread in the change of prostate V100% with source positioning errors across the 16 participating departments. One department failed the prostate V100% ≥ 90% criteria for a shift of even 1 mm, while others still passed these criteria even for shifts of up to 6 mm in each direction.

**FIGURE 3:**
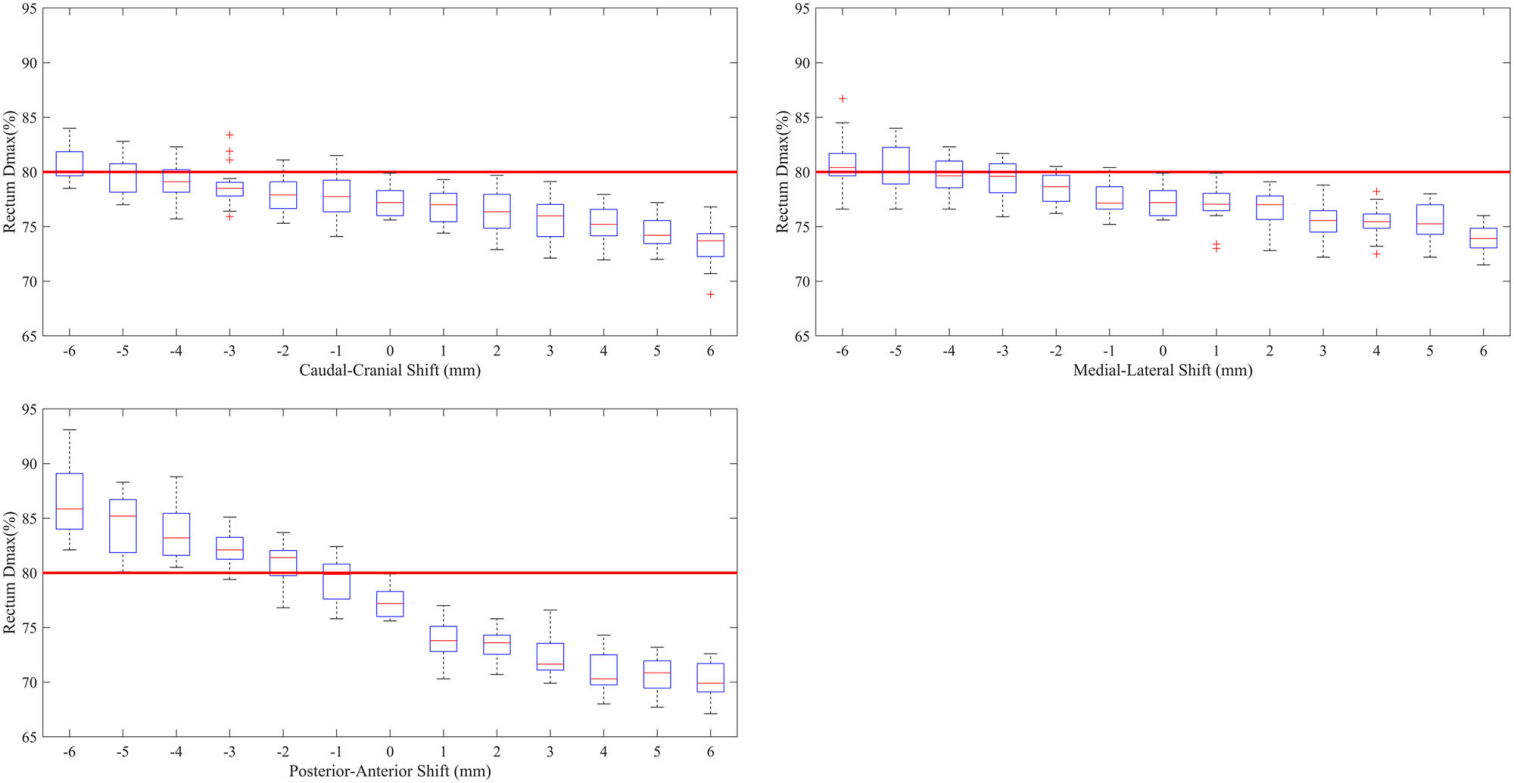
(A) Change in rectum *D*_max_ DVH metric as a function of source positioning error in the caudal (negative) and cranial (positive) direction, (B) The medial (negative) and lateral (positive) direction, and (C) the posterior (negative) and anterior (positive) direction. Red lines within the boxes represent the median across all 16 departments, the boxes represent the interquartile range, whiskers represent the minimum and maximum, red crosses represent the outliers. The thick solid red line represents the tolerance of 80% for the rectum *D*_max_ used to determine the source tracking error threshold

Figure 3 shows the effect of source positioning errors on the rectum *D*_max_ for all 16 departments. As expected, the sensitivity of the rectum *D*_max_ was largest to source positioning errors in the posterior direction. An increase in rectum *D*_max_ was also observed for source position errors in the medial and caudal directions. As for the prostate V100%, there was a significant spread in the rate of change of the rectum *D*_max_ across the participating departments. Some departments failed the rectum *D*_max_ < 80% criteria for a shift only 1 mm in the caudal, medial, and posterior directions, while others required source position errors of up to 4–6 mm in these directions to fail this planning metric.

**FIGURE 4:**
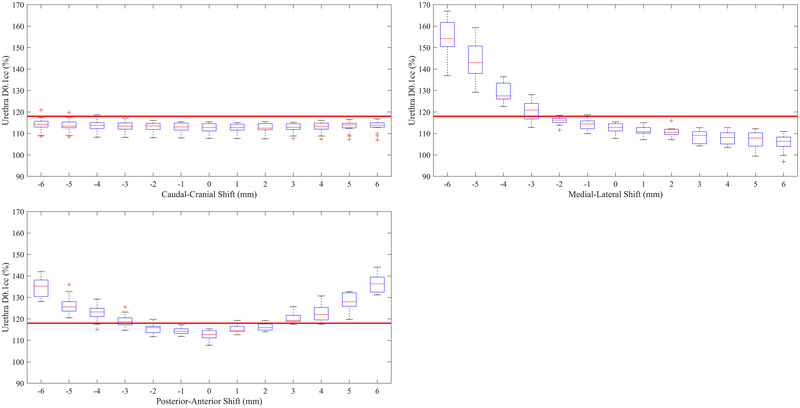
(A) Change in urethra D0.1cc DVH metric as a function of source positioning error in the caudal (negative) and cranial (positive) direction, (B) the medial (negative) and lateral (positive) direction, and (C) the posterior (negative) and anterior (positive) direction. Red lines within the boxes represent the median across all 16 departments, the boxes represent the interquartile range, whiskers represent the minimum and maximum, red crosses represent the outliers. The thick solid red line represents the tolerance of 118% for the urethra D0.1cc used to determine the source tracking error threshold

The sensitivity of the urethra D0.1cc to source positioning errors is shown in Figure 4. The urethra was not significantly sensitive to source positioning errors in the caudal or cranial direction with only a single department failing the urethra D0.1cc < 118% criteria for shifts up to 6 mm. The urethra D0.1cc was most sensitive to source positioning errors in the medial direction, with all departments failing the acceptability criteria for a shift of 4 mm in this direction. Finally, the urethra D0.1cc was somewhat sensitive to source positioning errors in the posterior and anterior directions, with the median D0.1cc increasing above the acceptability level of 118% for shifts of 3 mm in these directions. As for the prostate V100% and rectum *D*_max_, there was a considerable spread in the sensitivity of the urethra D0.1cc to source positioning errors in all directions.

### 3.3 Plan robustness parameter and correlation to in‐vivo source tracking error thresholds

**FIGURE 5:**
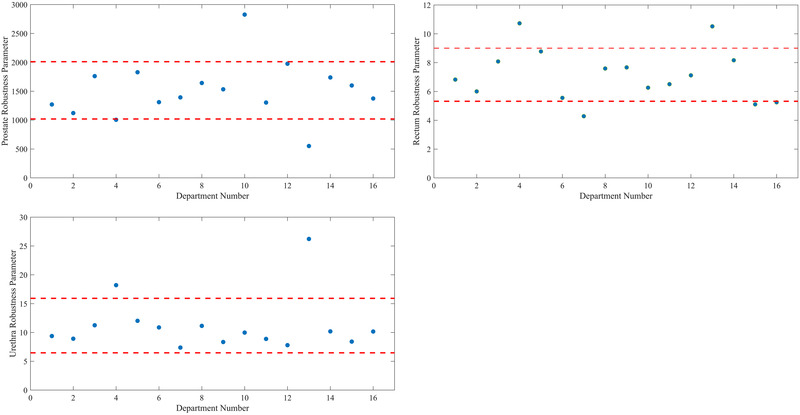
(A) Prostate robustness parameter for each of the 16 departments, (B) rectum robustness parameter, (C) urethra robustness parameter. Red dashed lines represent ±1 standard deviation from the mean of the 16 departments

The average prostate robustness parameter was found to be 1515.0 ± 495.9 (1 standard deviation), and the average rectum and urethra robustness parameters were found to be 7.2 ± 1.8 and 11.2 ± 4.7, respectively. Plots of the robustness parameters for prostate, rectum, and urethra for all 16 departments are shown in Figure 5A‐C, respectively.

From Figure 5A, two departments are observed as outliers (outside of the ±1 standard deviation limits). These departments also corresponded to those with the largest, and smallest sensitivity of the prostate V100% to source positioning errors in Figure 2. Similar results were also observed for the rectum and urethra contours.

To test the correlation of the robustness parameter to the DVH metrics sensitivity to source positioning errors, a Pearson's correlation coefficient was calculated for correlation against the change in prostate V100%, rectum *D*_max_, and urethra D0.1cc, in each source position error direction. Statistical significance was calculated for this correlation (ɑ < 0.05), and the results are summarized in Table 3. There was excellent correlation found for all DVH metric or direction combinations to the robustness parameter, with the lowest correlation coefficient calculated being 0.81. All correlations were also found to be statistically significant.

**TABLE 3:** Pearson's correlation coefficient (*r*), and statistical significance (*ɑ*) for the correlation of the sensitivity of the DVH metric to source positioning errors against the robustness parameter derives in Equation 1

DVH metric	Source position error direction	*r*	*ɑ*
Prostate V_100_%	Caudal‐Cranial	0.94	0.02
Prostate V_100_%	Medial‐Lateral	0.88	0.03
Prostate V_100_%	Posterior‐Anterior	0.81	0.02
Urethra D_0.1cc_	Caudal‐Cranial	0.91	0.03
Urethra D_0.1cc_	Medial‐Lateral	0.97	0.02
Urethra D_0.1cc_	Posterior‐Anterior	0.93	0.01
Rectum *D*_max_	Caudal‐Cranial	0.92	0.02
Rectum *D*_max_	Medial‐Lateral	0.83	0.04
Rectum *D*_max_	Posterior‐Anterior	0.97	0.02

## 4 DISCUSSION

The aim of this study was to examine the effect of departmental planning techniques on appropriate in‐vivo source tracking error thresholds and determine if a single in‐vivo source tracking error threshold would be appropriate for the same patient anatomy. From Figures 2, 3, 4, it can be seen that a single in‐vivo source tracking error threshold could not be applied to all departments, even for the same patient's anatomy. There is a significant spread in the sensitivity of the prostate V100%, rectum D_max_, and urethra D0.1cc DVH criteria to source positioning errors, evidenced by the large interquartile ranges in the box‐whisker plots from Figures 2, 3, 4. Additionally, as found by Poder et al. in a previous study,^13^ the appropriate in‐vivo source tracking error threshold also depends heavily on the end‐point (target coverage vs. organ at risk dose), and the direction of the source positioning error.

As seen in Figure 5A–C, the value of the calculated robustness parameter varies widely depending on the contour of interest. However, to produce a robust plan, the robustness values for each contour must all be minimized. Future studies will focus on the interplay between these values, and aim to determine optimal methods to produce a global robustness minimum for HDR prostate BT plans.

As also seen in Figure 5A–C, departments 4 and 13 were observed to have the smallest prostate robustness parameter value, but the largest rectum and urethra robustness parameter values. Interrogation of these treatment plans showed a well‐covered prostate contour with the 100% isodose line, and OARs that were close to their dose limits. Additionally, the large weighted catheters in these plans were located closer to the rectum and urethra as compared to other department's plans. Conversely, department 10 had the largest prostate robustness parameter value, and average rectum and urethra robustness parameter values. This treatment plan was also the outlier in Figure 2A–C, not quite meeting the prostate V100% coverage goal whilst respecting the OAR dose limits. The large weighted catheters in this plan were located closer to the prostate periphery, and therefore, small shifts of these catheters resulted in a faster loss of prostate coverage, and less effect on the OARs, relative to the other plans included in this study.

To examine the effect of treatment plan characteristics on the sensitivity of DVH metrics to source positioning errors, plan characteristics listed in Table 2 (number of catheters, number of dwell positions, and TRAK) along with the BTPS used to create the treatment plan were tested for correlation against their sensitivity to source tracking error thresholds. To achieve this, the Pearson's correlation coefficient was again calculated. The maximum Pearson's correlation coefficient calculated for any of these combinations was found to be 0.53, and therefore, no strong correlation was found between any of these treatment plan characteristics and the sensitivity of these plans to source positioning errors. Consequently, the results of this study indicate that the most reliable predictor of plan robustness in HDR prostate BT is dwell position weights and their minimum distance to contour surfaces.

From Table 3, it can be seen that the robustness parameter given in Equation 1 may be used to predict a HDR prostate BT plan's robustness to source positioning errors. This parameter may be investigated in future studies to determine the feasibility of incorporating the parameter into the inverse optimisation phase of the treatment planning process to produce plans that are more robust to source positioning errors, as has been performed in the EBRT community.^17^, ^18^ Incorporating plan robustness into the inverse optimisation process may also ease the burden on in‐vivo source tracking devices, which have so far been shown to have accuracy comparable to what may be considered an in‐vivo source tracking error threshold.^6^ By making plans more robust to source positioning errors, both sensitivity and specificity of in‐vivo source tracking devices in detecting clinically relevant source positioning errors may be improved.^6^ An approach, such as this, may be even more valuable in the context of HDR prostate BT plans treating the entire prostate plus a boost to the dominant intraprostatic lesion(s),^19^ and even more so in the context of focal treatments to these lesions within the prostate.^20^

The effect of prostate motion relative to planned positions in stereotactic ablative body radiotherapy (SABR) has also been studied.^21^ In this study, Hewson et al. showed that prostate motion relative to its’ planned position can occur by up to 17 mm. If not corrected for via multileaf collimator (MLC) tracking or gating, this motion can result in significant underdosage of the PTV and/or overdosage of surrounding OARs. The amount of PTV underdosage and OAR overdosage in prostate SABR was found to be similar to that found in this study for geometrical errors of the same magnitude. Technological evolution through gating and MLC tracking was found to improve the delivered dose distribution in prostate SABR, indicating that technological evolution could also be implemented in HDR prostate BT to improve treatment delivery.

One limitation of this study is that only a single patient dataset, contoured by a single radiation oncologist was used. While it is widely accepted that both intra‐ and interobserver contouring variability contributes significantly to the total combined uncertainty in HDR prostate BT treatment planning,^22^ the aim of this study was to examine only the effect of departmental planning technique on source positioning error thresholds, and therefore, a single set of radiation oncologist contours was used. For the same reason, only a single patient dataset was used. The effect of patient variation on deriving appropriate in‐vivo source tracking error thresholds has been investigated in a previous study.^13^

Another limitation is that only the three most heavily catheters in the treatment plans were considered for source positioning errors. From the discussion on the robustness parameter, it is clear that it is not only the relative weight of a catheter within a treatment plan that contributes toward robustness, but also the proximity of its dwell positions to contour surfaces. Future studies will focus on the robustness of individual catheters within treatment plans, and how the robustness of individual catheters may affect the sensitivity of DVH metrics to source positioning errors.

## 5 CONCLUSIONS

From this study, it was found that a single HDR prostate BT in‐vivo source tracking error threshold cannot be applied across multiple departments, even for the same patient anatomy. There was wide variation in appropriate in‐vivo source tracking error thresholds among the 16 participating departments, ranging from 1 to 6 mm. This may be attributed to departmental planning and catheter placement strategies significantly impacting plan robustness to these source positioning errors.

The most reliable predictor of plan robustness in HDR prostate BT was found to be dwell position weights and their minimum distance to contour surfaces. A robustness parameter was, therefore, derived using these factors, and found to correlate with the sensitivity of plans to source positioning errors. The burden on in‐vivo source tracking devices may, therefore, be eased in future applications by incorporating HDR prostate BT plan robustness during the plan optimisation phase, improving the sensitivity and specificity of in‐vivo source tracking devices to high‐risk failure modes in HDR prostate BT treatments.

## CONFLICT OF INTEREST

The authors have no conflict of interest or funding sources to report.
